# Mechano-Enzymatic Degradation of the Chitin from Crustacea Shells for Efficient Production of N-acetylglucosamine (GlcNAc)

**DOI:** 10.3390/molecules27154720

**Published:** 2022-07-23

**Authors:** Xinjun Yu, Zengchao Jiang, Xiaodan Xu, Changyi Huang, Zheyi Yao, Xiao Yang, Yinjun Zhang, Dongsheng Wang, Chun Wei, Xuwei Zhuang

**Affiliations:** 1 Key Laboratory of Bioorganic Synthesis of Zhejiang Province, College of Biotechnology and Bioengineering, Zhejiang University of Technology, No. 1, Gongda Road, Huzhou 313200, China; xjyu@zjut.edu.cn (X.Y.); caco3atm@126.com (Z.J.); xuxiaodan7@163.com (X.X.); hcy929390419@gmail.com (C.H.); yzy1738641063@163.com (Z.Y.); yangx1787426@163.com (X.Y.); zhangyj@zjut.edu.cn (Y.Z.); 2 Institute of Biological Resources, Jiangxi Academy of Sciences, Nanchang 330096, China; 3 Zaozhuang Chengmei Environmental Engineering Consulting Co., Ltd., Linyi 276000, China; zhuangxuwei@163.com

**Keywords:** chitinase, shrimp and crab shell, N-acetylglucosamine, mechanoenzymology, heterologous expression

## Abstract

Chitin, the second richest polymer in nature, is composed of the monomer N-acetylglucosamine (GlcNAc), which has numerous functions and is widely applied in the medical, food, and chemical industries. However, due to the highly crystalline configuration and low accessibility in water of the chitin resources, such as shrimp and crab shells, the chitin is difficult utilize, and the traditional chemical method causes serious environment pollution and a waste of resources. In the present study, three genes encoding chitinolytic enzymes, including the N-acetylglucosaminidase from *Ostrinia furnacalis* (*OfHex1*), endo-chitinase from *Trichoderma viride* (*TvChi1*), and multifunctional chitinase from *Chitinolyticbacter meiyuanensis* (*CmChi1*), were expressed in the *Pichia pastoris* system, and the positive transformants with multiple copies were isolated by the PTVA (post-transformational vector amplification) method, respectively. The three recombinants OfHex1, TvChi1, and CmChi1 were induced by methanol and purified by the chitin affinity adsorption method. The purified recombinants OfHex1 and TvChi1 were characterized, and they were further used together for degrading chitin from shrimp and crab shells to produce GlcNAc through liquid-assisted grinding (LAG) under a water-less condition. The substrate chitin concentration reached up to 300 g/L, and the highest yield of the product GlcNAc reached up to 61.3 g/L using the mechano-enzymatic method. A yield rate of up to 102.2 g GlcNAc per 1 g enzyme was obtained.

## 1. Introduction

With the development of global fisheries, abundant processed aquatic products have entered people’s lives, and crustaceans, such as shrimp, crabs, lobsters, etc., account for a large proportion. Statistics from the World Food and Agriculture Organization in 2020 shows that approximately 9.3 billion tons of shrimp are consumed annually, and Asian countries account for 89.6% of the world’s consumption [1]. Shrimp meat consumed by human only account for 52–55% of their body weight [2], so that approximately 4.3 billion tons of crustacean waste, including the cephalothorax and shells, are produced annually. Except for a small part of the crustacean waste being used as feed and fertilizer, the left is discarded, landfilled, or incinerated, thus resulting in environmental problems, such as peculiar smell, bacterial growth, soil, water pollution, etc. [3,4]. Moreover, since shrimp and crab shells are composed of chitin (20–25%), protein (about 40%), and calcium carbonate (about 30%), chitin can be extracted from these crustaceans wastes and used in chemical, medical, and other fields [5].

Chitin is the second abundant biopolymer with the monomer N-acetylglucosamine (GluNAc) connected through the beta-1,4-glycosidic bond. Natural chitin has different crystal structures, which can be divided into α-chitin, β-chitin, and γ-chitin, with the layers parallelly, antiparallelly, and parallel–antiparallelly connected by hydrogen bonds, respectively [6]. Moreover, chitin is tightly combined with protein and calcium carbonate in nature. These structural features bring about the high crystallinity and insolubility of chitin, so that it is difficult to be utilized. Traditionally, the extraction and degradation of chitin were performed using chemical methods, such as strong acid and alkali treatments [7,8]. However, these chemical methods cause severe environmental issues. Thus, using chitinases to treat chitin, with mild reaction conditions and less pollution, has become an ideal strategy [9].

Chitinase, which belongs to the glycoside hydrolase (GH) family, can degrade beta-1,4-glycosidic bonds and break the polysaccharide chain, in order to obtain the molecules with lower degree of polymerization. According to their catalysis positions, chitinases can be divided into endo-chitinase (EC 3.2.1.14), exo-chitinase (EC 3.2.1.200 and EC 3.2.1.201), and N-acetylglucosaminidase (NAGase) (EC 3.2.1.52) [10]. The chitinases are widely distributed in microorganisms, insects, and plants, and they have numerous functions in the biological world. Although chitinase is distributed widely in nature and has multiple biological functions, there is still a long way to go, in order to apply it in industry. Usually, chitin can be degraded by chitinases through two steps, in which the initial cleavage of the chitin polymer via endo- or exo-chitinase into chitin oligosaccharide and second cleavage to GlcNAc by NAGase. The chitinase from *Trichoderma viride* LTR-2 (TvChi1), which has a high homology with other endo-chitinases in GH18, belongs to an endo-chitinase and can degrade chitin into oligosaccharides with high efficiency [11,12]. Exo-chitinase catalyzes the beta-1,4-saccharidic bond between the second and third GlcNAc residues to produce chitobiose. The NAGase catalyzes the beta-1,4-glycosidic bond at the end of the polysaccharide chain to get GlcNAc. The chitinase from *Ostrinia furnacalis* (OfHex1) is a NAGase belonging to GH20, which can degrade chitin oligosaccharides into GlcNAc efficiently [13]. In recent years, some chitinases were found to have more than one catalysis position and are referred to as multifunctional chitinase. These chitinases usually possess two or all catalytic activities of the endo-, exo-chitinase, and NAGase [14]. For example, the chitinase from *Chitinolytic meiyuanensis* SYBC-H1 (CmChi1) is a multifunctional chitinase belonging to the GH18 family. The CmChi1 shows the catalytic activity of endo-, exo-chitinase, and NAGase simultaneously when colloidal chitin was used as a substrate; high purity of GlcNAc was obtained, with a yield of nearly 100%. 

However, the layer structure of the chitin and the hydrogon bonds between layers make chitin insoluble and hard to degrade enzymatically. Thus, the pretreatment of chitin, mainly composed from physical, chemical, and biological methods, is essential for chitin degradation [15]. The physical method increases the specific surface area and pore size and reduces the crystallinity and polymerization degree through mechanical pulverization, radiation, and so on. The mechano-enzymatic method is one of the most emergent physical pretreatment methods, and the substrate is grinded and extruded by a ball miller or twin-screw extruder in this method [16]. There have been many successful reports of mechano-enzymatic catalytic reactions. The solvent free mechano-enzymatic method, combined with immobilized *Candida antarctica* lipase B (CALB), can resolve racemic chiral amines with high enantioselectivity and regioselectivity [17]. Meanwhile, the mechano-enzymatic method can solve the solubility problem in cellulose or chitin degradation [18]. Shawn et al. applied ball milling as a digestion container, in order to degrade α-cellulose enzymatically, which increases the saccharification rate, decreases enzyme loading, and reaches a yield of 100% [19]. It is demonstrated that ball milling can promote the treatment of weak acid to remove mineral and protein from shrimp and crab shells and degrade chitin into soluble products [20,21]. However, the single enzyme that was used in the mechano-enzymatic reaction has low stability and degrading rates. Chitin degradation with a multi-enzyme system based on the mechano-enzymatic technology has never been reported.

In this study, the NAGase gene from *O. furnacalis* (*OfHex1*) and chitinase genes from *T. viride* (*TvChi1*) and *C. meiyuanensis* (*CmChi1*) were heterologously expressed in the *Pichia pastoris* system, respectively. The recombinant TvChi1, OfHex1, and CmChi1 were purified, and the TvChi1 and OfHex1 were characterized to obtain the optimal reaction condition. Finally, the three recombinant chitinases were combined to degrade the chitin derived from Crustacea shells into GlcNAc through mechano-enzymatic technology. This research provides a foundation for marine biological resource utilization and GlcNAc production at the industrial scale.

## 2. Materials and Methods

### 2.1. Chemicals

Restriction enzymes, T4 DNA ligase, and DNA polymerase were purchased from Takara Biomedical Technology Co., Ltd. (Beijing, China). EasyPure Plasmid Miniprep Kit was purchased from Tsingke Biotechnology Co., Ltd. (Beijing, China). TIANquick Midi Purification Kit was purchased from Tiangen Biotech Co., Ltd. (Beijing, China). Rapid Yeast Genomic DNA Isolation Kit, yeast powder, peptone, kanamycin, ampicillin, G418 sulfate, and YNB (yeast nitrogen base, without amino acid and ammonium sulfate) were purchased from Sangon Biotech Co., Ltd. (Shanghai, China). GlcNAc was purchased from Aladdin Bio-Chem Technology Co., Ltd. (Shanghai, China). All chemicals used were of analytical grade.

### 2.2. Construction of Pichia Pastoris Strains Heterologously Expressing Chitinase Genes

The optimized genes *OfHex1* (NCBI accession number: ABI81756.1), *TvChi1* (NCBI accession number: ABR27743.1), and *CmChi1* (NCBI accession number: ATN39892.1) were cloned into the expression plasmid pPIC9K, respectively [13]. The primer pair 5-AOX (GACTGGTTCCAATTGACAAGC) and 3-AXO (GGCAAATGGCATTCTGACAT), with *Sna*B Ⅰ and *Not* Ⅰ as the restriction sites, was used in this study. The three chitinase genes was ligated with the plasmid pPIC9K at the same restriction site to form the recombinant plasmids pPIC9K-*OfHex1*, pPIC9K-*TvChi1*, and pPIC9K-*CmChi1,* respectively. The three recombinant plasmids were transformed into the competent *P. pastoris* GS115 cells, respectively. The recominant strains GS115/pPIC9K-OfHex1, GS115/pPIC9K-TvChi1, and GS115/pPIC9K-CmChi1 were obtained after G418 screening and confirmation of the exogenous chitinase genes existed in the recombinant strain by PCR technology. The PCR procedure: 98 °C for 5 min; 25 cycles of 98 °C for 10 s; 60 °C for 5 s; 72 °C for 10 s; 72 °C for 5 min.

### 2.3. Induced Expression of the Three Recombinant Chitinases

The recombinant *P. pastoris* strains GS115/pPIC9K-OfHex1, GS115/pPIC9K-TvChi1, and GS115/pPIC9K-CmChi1 were inoculated into YPD medium containing 250 μg/mL G418 sulfate and cultured at 200 g and 30 °C for 16 h. Then, a volume of 1mL seed culture was inoculated into BMGY medium containing 250 μg/mL G418 sulfate and cultured for 20 h. The culture was centrifuged at 5000× *g* and 4 °C for 5 min, and the cells were then transferred into the BMMY medium. For gene expression induction, 1% methanol was added into the BMMY at an interval of 24 h for 96 h. The induced protein was centrifuged at 8000× *g* and 4 °C for 10 min, and the supernatant was collected as the crude enzyme for further analysis.

### 2.4. Enzymatic Activity Assay

The colloidal chitin was used as substrate to determine enzymatic activity. The 500 μL reaction system contained 66 μL colloidal chitin (10%, *w*/*v*), 334 μL buffer (pH 5.0), and 100 μL crude chitinase. The reaction was performed by shaking at 50 °C for 2 h and stopped by boiling at 100 °C for 10 min. The reacting product was centrifuged at 12000× *g* for 5 min, and the reducing sugar content was determined by DNS method [22]. One unit (U) of the chitinase activity is defined as the amount of enzyme required to release 1 μmoL of reduced sugar per minute. The protein concentration was measured by standard Bradford assay.

### 2.5. Purification of the Recombinant Chitinases TvChi1 and CmChi1

The chitinases TvChi1 and CmChi1 were purified by chitin affinity adsorption, respectively, based on the method described by Zhang et al., with minor revisions [23]. A volume of 1 mL colloidal chitin (1%) was added into 2 mL crude enzyme solution and incubated at 0 °C for 30 min. After centrifugation at 8000× *g* and 4 °C for 5 min, the supernatant was discarded. The precipitation was washed by 10 mL sodium chloride solution (1 M) to remove heterogeneous proteins, which was repeated 3 times. Then, a volume of 10 mL pre-cooled deionized water was used to wash the precipitation. After resuspension with phosphoric acid buffer (0.2 M, pH 5.0), it was incubated at 37 °C for 4 h. When chitin was degraded into soluble sugar, the chitinase was released into solution and collected in supernatant by centrifugation. After ultrafiltration, the concentrated chitinase was collected and stored at −20 °C for further analysis. The purity of the recombinant chitinase was detected by SDS-PAGE, and the protein concentration was determined by Bradford method using bovine serum albumin (BSA) as a standard.

### 2.6. Characterizations of the Recombinant Chitinases TvChi1 and CmChi1

The optimal temperature for recombinant chitinase was determined in the range of 20–70 °C. The reaction system and activity analysis were performed as described above. The reaction system was shaken in a metal bath for 2 h and then bathed in boiled water to end reaction. The purified enzyme was boiled in water for 10 min before a reaction as the blank control group. The enzyme activity in optimal temperature was defined as 100%. For temperature stability, the purified enzyme was incubated in the range of 20–70 °C for 3 h, respectively, and its activity was determined. The inactivated enzyme before reaction was set as the control group. The specific enzyme activity, stored at 4 °C under the same condition, was defined as 100%.

The optimal pH for recombinant chitinase was determined at a range of pH 3.0–9.0. The reaction was set at different pHs and 50 °C for 2 h and stopped by boiling for 10 min. The enzyme activity at the optimal pH was defined as 100%. For pH stability, the purified enzyme was incubated at a range of pH 3.0–9.0 for 3 h, and their activities were determined. The specific enzyme activity without pH treatment was defined as 100%.

Different metal ions, including Al^3+^, Ca^2+^, Co^2+^, Cu^2+^, Fe^3+^, K^+^, Mg^2+^, Mn^2+^, Na^+^, Ni^+^, and Zn^2+^, with two final concentrations of 1mM and 5mM, were used to measure the effects of metal ions on recombinant chitinases. After the chitinase was incubated with different metal ions at 4 °C for 3 h, the activity was analyzed as described above. The enzymatic activity without any metal ion treatment was defined as 100%.

### 2.7. Mechano-Enzymatic Reaction

The mechano-enzymatic reaction was composed of LAG and Raging. Shrimp and crab shells with protein and calcium removed were crushed into powder as substrate. The reaction system was composed of 5 g substrate, 5 g chitinase powder (~2% content), 80 g grinding beads (2 mm and 304 stainless steel), and 10 mL sodium acetate buffer (pH 5.0) in the ball mill tank, with 304 stainless steel as material. The ball mill tank was loaded into planetary ball mill, with grinding at 451 g for 30 min. Then, the sample was incubated at 53 °C for 12 h. The treated sample was stored at −80 °C until analysis.

For optimizing the mechano-enzymatic reaction, 1.0, 2.5, 5.0, 7.5, and 10.0 g substrate with 5 g chitinase powder were fixed to optimize the substrate concentration. Moreover, the material-to-bead ratio was optimized by a range of 20, 40, 60, 80, 100, 120, and 140 g grinding beads, with the material content fixed. For LAG time, the reaction was performed for 10–60 min, respectively. The Raging time and temperature were optimized by the same method, at a range of 30–60 °C and 4–12 h, respectively.

### 2.8. Analysis of the Product GlcNAc

The sample was dried by vacuum drying at 75 °C, crushed into powder, and sieved to remove grinding beads. An amount of 0.15 g sample was dissolved in 1 mL water and then centrifuged in 12,000× *g* for 1 min to remove the insoluble. A total of 200 μL supernatant, 400 μL NaOH (0.3 M), and 400 μL 1-phenyl-3-methyl-5-pyrazolone (PMP, 0.5 M in methanol solution) were mixed and incubated at 70 °C for 30 min. After the system was cooled down naturally, 400 μL HCl (0.3 M) was added and extracted with chloroform 3 times, with the chloroform phase discarded. Then, the sample was centrifuged at 12,000× *g* for 1 min, and the supernatant was loaded to a C18 column equipped in the HPLC system. The detection wavelength and column temperature were set at 245 nm and 25 °C. 

### 2.9. The Multi-Enzyme System for GlcNAc Production

The multi-enzyme system, composed of 6 mg OfHex1, 6 mg TvChi1, and 8 mg CmChi1, combining with 10 g chitin powder and 80 g grinding bead, were added into the ball mill tank. The LAG reaction was at 500 g and pH 5.0 for 30 min. Then, for Raging, the sample was incubated at 45 °C for 20 h. The amount of GlcNAc monomer was determined by HPLC, as described above.

## 3. Results

### 3.1. Heterologous Expression of Three Recombinant Chitinases

**Figure 1:**
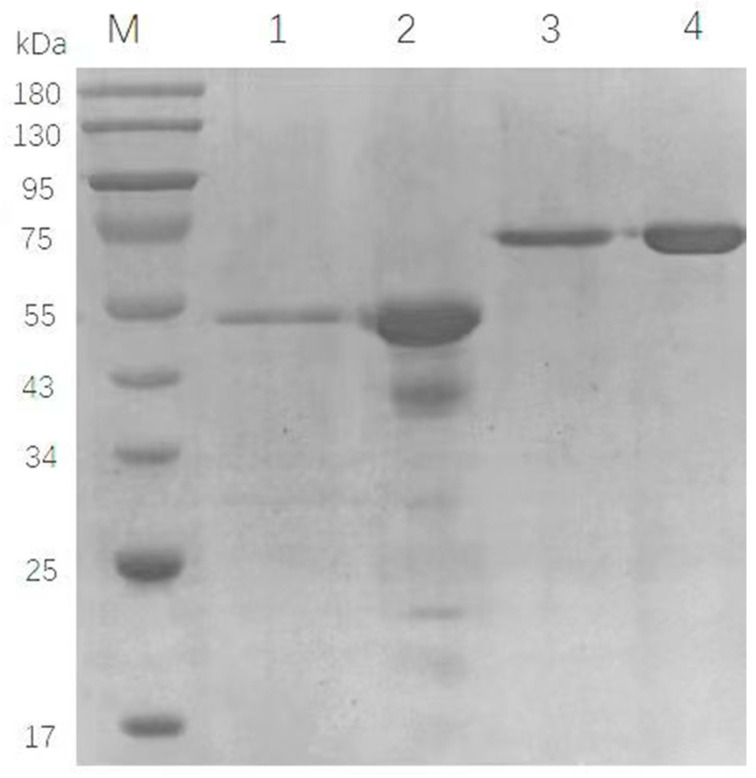
SDS-PAGE results of the purified TvChi1 and CmChi1. Lanes 1–4 are purified TvChi1, crude enzyme solution of TvChi1, purified CmChi1, and crude enzyme solution of CmChi1, respectively.

According to amino acid sequences, the theoretical molecular weights of OfHex1, TvChi1, and CmChi1 were predicted at 68.0, 46.3, and 70.1 kDa, respectively. Then, the three chitinase genes were expressed in *P. pastoris*, and the molecular weights of the recombinants OfHex1, TvChi1, and CmChi1 were 48, 46, and 70 kDa, respectively, consistent with their theoretical values (Figure 1) [13,23]. Moreover, the enzymatic activities of crude OfHex1, TvChi1, and CmChi1 were 0.2 U/mL, 0.6 U/mL, and 0.7 U/mL, respectively, suggesting that these recombinant chitinases was successfully expressed in the *P. pastoris*. Thus, for a better application of these chitinase, the recombinant CmChi1 and TvChi1 were further purified and characterized for their application in chitin degradation.

### 3.2. Purification of the Two Recombinant Chitinases

As the *OfHex1* gene has been expressed and purified in *P. pastoris* GS115 and the recombinant OfHex1 has been characterized [13], in current study, the other two chitinase, the recombinant CmChi1 and TvChi1, were purified through the interactions between chitinase and chitin in the chitin affinity adsorption method [23]. As shown in Figure 1, the recombinant TvChi1 and CmChi1 were purified to a single band in SDS-PAGE analysis, thus indicating that the chitin affinity adsorption method was ideal for chitinase purification [23]. The specific activity for the purified recombinants TvChi1 and CmChi1 were 5.2 and 35.9 U/mg, with purification factors of 3.5- and 5.6-times, respectively (Table 1). However, the purification yields of recombinant TvChi1 and CmChi1 using the chitin affinity adsorption method were 10.2% and 13.2%, respectively, which was lower than those from the His-tags and Ni column purification [23,24,25]. This might be due to the relatively small specific surface area of the colloidal chitin, which could not combine with as much chitinase as the Ni column. Moreover, the weak affinity adsorption may be the other reason, thus resulting in the elution of the target protein during the washing process [23].

### 3.3. Characterizations of the Two Recombinant Chitinases

**Figure 2:**
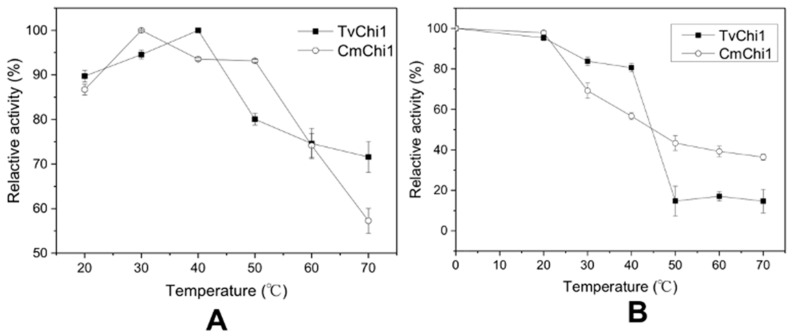
Effect of temperature on activities of the TvChi1 and CmChi1. (**A**) The optimal temperature for TvChi1 and CmChi1; (**B**) the temperature stability for TvChi1 and CmChi1. Data are given as means ± standard deviation, *n* = 3.

For a better performance in chitin degradation, the recombinant TvChi1 and CmChi1 were firstly characterized. As shown in Figure 2A, the optimal temperatures for TvChi1 and CmChi1 were 40 °C and 30 °C, respectively, with the same change curve as the previous study [23]. At the temperature range of 20–40 °C, the activity of TvChi1 reached 90% of the optimal and up to 70% at a range of 4–40 °C for 3 h. For the CmChi1, the activity was kept up to 90% of the optimal, at the temperature range of 30–50 °C, and even up to 70% at the range of 4–30 °C for 3 h. Moreover, the CmChi1 activity remained up to 40%, even when the temperature reached 70 °C (Figure 2B). 

**Figure 3:**
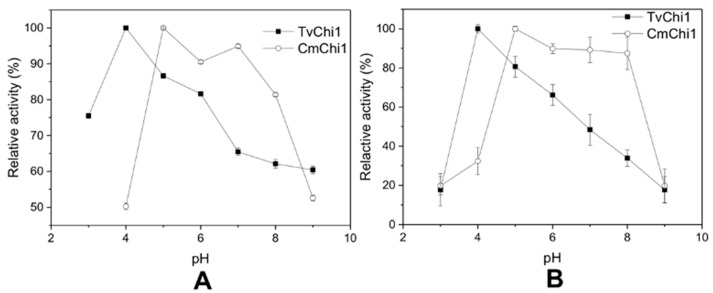
Effect of pH on activity of the TvChi1 and CmChi1. (**A**) The optimal pH for TvChi1 and CmChi1; (**B**) the pH stability for TvChi1 and CmChi1. Data are given as means ± standard deviation, *n* = 3.

The effects of pH on recombinant TvChi1 and CmChi1 were also analyzed. The optimal pH for TvChi1 and CmChi1 were 4.0 and 5.0, respectively (Figure 3A). As shown in Figure 3B, TvChi1 was stable at a range of pH 4.0–6.0, and its residual activity still remained up to 60% below pH 3.0 or above 7.0. CmChi1 was stable at a range of pH 5.0–8.0, with up to 80% of the original activity remained. However, when pH reached 3.0–4.0 or above 8.0, only 20–30% of the original activity for CmChi1 remained. These results indicate that the TvChi1 and CmChi1 are optimal in acidic or neutral conditions, which is the environment of chitin degradation. 

**Figure 4:**
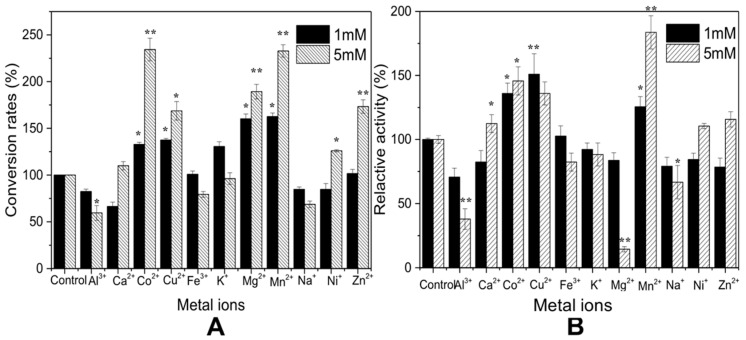
Effects of metal ions on the activity of TvChi1 and CmChi1. (**A**) TvChi1; (**B**) CmChi1. Data are given as means ± standard deviation, *n* = 3; ** *p*-value < 0.01, * *p*-value < 0.05.

Metal ions act as cofactors for enzymatic reactions, in order to help enzymes bind to substrates or catalyze efficiently, while some of them inhibit enzyme reactions. In this study, different kinds of metal ions, with two concentrations (high and low), were analyzed for their effects on recombinants TvChi1 and CmChi1 (Figure 4). For TvChi1 (Figure 4A), 1 mM of Co^2+^, Cu^2+^, K^+^, Mg^2+^, and Mn^2+^ promoted enzymatic activity, among which Mg^2+^ and Mn^2+^ increased enzymatic activity by 60% and 63%, compared to the control group without any metal ion treatment. By contrast, Al^3+^, Ca^2+^, Na^+^, and Ni^+^ inhibit enzymatic activity, among which Ca^2+^ showed the strongest suppression effect, with only 66% of activity remaining after treatment. At a concentration of 5 mM, Co^2+^, Cu^2+^, Mg^2+^, and Mn^2+^ promoted enzymatic activity obviously, among which Co^2+^ and Mn^2+^ increased enzymatic activity by 234 and 233%, compared to the control group. Ca^2+^, Ni^+^, Zn^+^, Al^3+^, and Na^+^ showed inhibitive effects on enzymatic activity, and the inhibitive effects of Al^3+^ were more obvious, with only 60% of activity remaining after treatment. For CmChi1 (Figure 4B), 1mM of Co^2+^, Cu^2+^, and Mn^2+^ had positive effects on the enzymatic activity, among which Cu^2+^ increased enzymatic activity by up to 151%, compared to the control group. A concentration of 5mM Co^2+^, Cu^2+^, and Mn^2+^ increased their promotive effects, compared to the 1 mM condition, and Mn^2+^ increased the enzymatic activity by 184% to the control group. Moreover, the higher concentration also enforced the suppression effects of Al^3+^, Mg^2+^, and Na^+^, and 5 mM Mg^2+^ had the most inhibitive effect, with only 14% of enzymatic activity remaining after treatment.

### 3.4. Optimization of Reaction Parameters for GlcNAc Production

**Figure 5:**
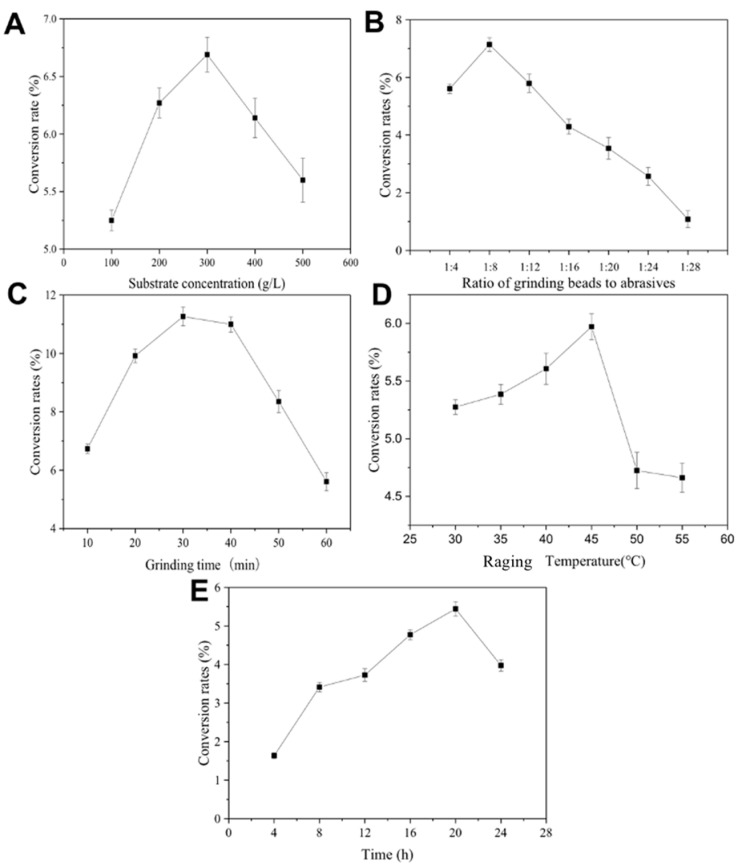
Optimizations of liquid-assisted grinding (LAG) and Raging for GlcNAc production. (**A**) Substrate concentration; (**B**) ratio of beads to abrasives; (**C**) grinding time; (**D**) raging temperature; (**E**) reaction time. Data are given as means ± standard deviation, *n* = 3. The conditions: (**A**) beads 80 g, chitinase 5 g, 10 mL sodium acetate buffer with pH 5.5, 451 rpm grinding for 30 min, and Raging at 53 °C for 12 h, and different concentrations of chitin powder were set; (**B**) chitin powder 5 g, chitinase 5 g, and 10 mL sodium acetate buffer with pH 5.5, 451 rpm grinding for 30 min, and Raging at 53 °C for 12 h, different concentrations of beads were set; (**C**) chitin powder 5 g, chitinase 5 g, 10 mL sodium acetate buffer with pH 5.5, beads 80 g, 451 rpm grinding, and Raging at 53 °C for 12 h, and different grinding times were set; (**D**) chitin powder 5 g, chitinase 5 g, 10 mL sodium acetate buffer with pH 5.5, beads 80 g, 451 rpm grinding for 30 min, and Raging for 12 h, and different Raging temperatures were set; (**E**) chitin powder 5 g, chitinase 5 g, 10 mL sodium acetate buffer with pH 5.5, beads 80 g, 451 rpm grinding for 30 min, and Raging at 53 °C, and different Raging times were set.

As the reactions were processed in LAG and Raging, various parameters can be optimized to increase the conversion rate for GlcNAc production. As shown in Figure 5, it was demonstrated that 300 g/L of substrate, 1:8 of grinding beads to abrasives, 30 min of grinding time, and 20 h and 45 °C of Raging time and temperature were optimal for the conversion rate from chitin to GlcNAc production. Under the optimal conditions, up to 61.3 g/L of GlcNAc, with a productivity of 102.2 g GlcNAc per 1 g enzyme, was obtained.

## 4. Discussion

As the previous study reported, the *OfHex1* gene has been expressed in *P. pastoris*, which indicated that *P. pastoris* is an ideal system for recombinant chitinase expression, due to its excellent secretion activity and specific glycosylation modification [13]. Thus, we selected this system for producing the three recombinant chitinases, i.e., OfHex1, CmChi1, and TcChi1, for the degradation of chitin, in order to produce GlcNAc. The activity of the recombinant OfHex1 was similar with the previous report [13]. It is worth mentioning that the specific activity of the recombinant CmChi1 from the *P. pastoris* system (0.7U/mL) was nearly two times that from *E. coli* (0.4U/mL) [23]. Moreover, the characteristics of the recombinant chitinase expressed in *P. pastoris* were obviously different from those in other expression system. For example, the TvChi1 and CmChi1 derived from the *P. pastoris* system had higher thermal stability than those in *E. coli* (Figure 2) [23]. Thermal stability of chitinase was pivotal for enzymatic reaction because the further reactions were performed at relative high temperature conditions. The Mn^2+^ and Ni^+^, which were rich in 304 stainless steels as the main reactor materials, also had positive effects on the activity of the recombinants TvChi1 and CmChi1, suggesting that the recombinant chitinases working in the reactor might show better performance. The *P. pastoris* has excellent secretion activity and a specific glycosylation modification model for the recombinant protein; thus, the recombinant chitinases from this system are the ideal catalyst for the degradation of chitin for GlcNAc production. 

Due to the specific structural features, such as high crystallinity and insolubility, chitin is inaccessible and very difficult to use. Thus, pretreatment of chitin, mainly by physical, chemical, and biological methods, is essential for chitin degradation [15]. The physical pretreatment of chitin increases the specific surface area and pore size and reduces the crystallinity and polymerization degrees through mechanical pulverization, radiation, and so on. However, the liquid reaction system, in which chitin is not dissolved well, is not suitable for chitin utilization. Thus, in this study, the cycle of liquid-assisted grinding (LAG) under a water-less condition and Raging using three chitinases, with different functions as catalysts, were applied for the degradation of chitin, in order to produce GlcNAc. Various reaction factors, such as substrate concentration, ratio of beads to abrasives, grinding time, and Raging temperature and time, were systematically optimized for a higher conversion from chitin to GlcNAc. Surprisingly, up to 300 g/L of chitin (as substrate) was optimal for reaction, which was much higher than the highest solubility of chitin, even in the ionic liquid as cosolvent (about 40 g/L), with limited yield of GlcNAc. In the current study, 61.3 g/L of GlcNAc, with only a 20.4% conversion rate from the chitin substrate, was obtained, which overcomes the problem of chitin solubility and limit of GlcNAc yield, which calls for more improvements in the enzymatic performance and reaction process in future studies. Moreover, based on the concentrations of three purified recombinant chitinases, a total of about 0.6 g/L enzyme was applied in the reaction system, and a high yield rate of 102.2 g GlcNAc per 1 g enzyme was obtained, which was more economic and feasible, as well as higher than that of the results reported by Wang et al. [26]. 

## 5. Conclusions

The mechano-enzymatic method combined with complex recombinant chitinases is efficient in chitin degrading and GlcNAc production. The yield of 61.3 g/L GlcNAc has the potential for industrial application with less pollution, wasted resources, and reaction cycles. Chitinases with higher activities, direct evolution for activity improvement of chitinase, or further optimization of LAG and Raging process can lead to a higher yield.

## Figures and Tables

**Table 1:** Purification of TvChi1 and CmChi1.

	Steps	Total Protein (mg)	Total Activity (U)	Specific Activity (U/mg)	Purification Fold	Recovery Rate (%)
TvChi1	Crude enzyme	1112.4	5.8	5.2	1.0	100.0
	Purified enzyme	32.9	0.6	17.9	3.5	10.2
CmChi1	Crude enzyme	1153.8	7.4	6.4	1.0	100.0
	Purified enzyme	27.2	1.0	35.9	5.6	13.2

## Data Availability

Not applicable.

## Sample Availability

Samples of the compounds are not available from the authors.
